# Oral Tranexamic Acid Treats Papulopustular Rosacea by Improving the Skin Barrier: Correspondence

**DOI:** 10.1111/jocd.16788

**Published:** 2025-01-13

**Authors:** Hannah Verma, Brandon Block, Raphaella A. Lambert, Grace Rabinowitz, Nicholas Gulati, Benjamin Ungar

**Affiliations:** ^1^ Department of Dermatology Icahn School of Medicine at Mount Sinai New York New York USA

**Keywords:** DVT, inflammatory skin disease, papulopustular rosacea, rosacea, stroke, thrombosis, tranexamic acid

We recently read with interest the novel publication “Oral tranexamic acid treats papulopustular rosacea by improving the skin barrier” by Xu et al. [1] The authors describe their single‐centered randomized controlled trial (RCT) conducted at Xi'an Jiaotong University in Shaanxi Province, China, where patients in the experimental arm received a combined dose of oral tranexamic acid (TXA) 250 mg twice daily and 50 mg oral doxycycline. At the end of eight weeks, significant improvements were observed in clinical erythema, investigators' global assessment of rosacea, and patient‐self assessment of rosacea, without major adverse events, including those related to coagulopathy. This trial represents a significant step in the expansion of the currently limited arsenal of pharmacologic therapies for rosacea. The authors' findings show potential for oral TXA for the treatment of rosacea. While the authors took great care in following subjects' coagulation parameters over the course of 12 weeks, we believe that the long‐term safety of oral tranexamic acid in rosacea patients merits further discussion.

Although oral TXA has been shown to pose a limited risk of deep venous thrombosis (DVT) in patients with melasma, the evidence is lacking for patients with chronic inflammatory skin diseases like rosacea [2]. One case report has been published on this topic, in which a 37‐year‐old female patient with rosacea who previously failed first‐line treatment saw significant improvement after 8 weeks of triple therapy with oral propranolol, minocycline, and TXA (250 mg) [3]; however, the long‐term safety profile of oral TXA in patients with inflammatory skin disease has not been well established. The authors of this RCT were careful to follow patients for 1 month following discontinuation of TXA, but additional data on whether the therapeutic effect and safety profile of oral TXA persist beyond this time point is an important consideration for future studies. The chronic, recurrent, and relapsing nature of the disease could require long‐term use of oral TXA, which might increase the risk of thrombotic adverse events beyond the 12‐week duration studied in this RCT. In addition, patients in the experimental arm of this RCT received 50 mg of doxycycline daily, while patients in the control arm received only 40 mg; the difference in this dosing of doxycycline was not clear and could have potentially influenced the differences in measures of clinical response. We feel that further investigation into the long‐term efficacy and safety profile of oral TXA is warranted.

The literature suggests that rosacea itself may be linked to increased prevalence of thrombosis, potentially due to its systemic inflammatory nature. A prior case–control study conducted at Beth Israel Deaconess Medical Center found that patients with rosacea had increased adjusted odds of venous thromboembolism and DVT compared to healthy controls [4]. A separate 2023 study reporting an analysis of 27 patients with rosacea observed a significantly greater prevalence of genetic mutations related to increased platelet aggregation and decreased fibrinolytic activity [5]. Given these findings, dermatologists should carefully assess coagulation parameters and other thrombotic risk factors in rosacea patients prior to considering long‐term oral TXA therapy to reduce the risk of potential thromboembolism or stroke.

In examining the demographics of patients in the experimental arm of the RCT, we observed that the median reported age and mean BMI were 28 years and 20.61, respectively. Notably, several other studies have found rosacea to be associated with obesity [6, 7]. For example, 59% of patients in the COpenhagen ROsacea Cohort (*n* = 300) were either overweight or obese [7]. In addition, the onset of rosacea most commonly occurs at or after age 30 [8, 9]. Therefore, the generalizability of this trial should be considered, as neither arm of the trial may fully represent the global population of individuals suffering from rosacea, whose increased age and BMI, on average, could theoretically predispose to a greater risk of vascular adverse events than was observed in the study.

Preliminary studies with topical, micro‐needled, and intradermal TXA have also shown efficacy in reducing erythema in rosacea via inhibition of angiogenesis, without any risk of thrombosis [10, 11, 12]. Future clinical trials could compare the efficacy of oral TXA to these localized formulations to determine if the benefits of the former justify its potentially increased risk profile. Xu et al. demonstrate the effectiveness of oral TXA in their patient population, and it would be of great clinical value to have results from larger studies assessing longer‐term efficacy and safety of oral TXA in patients with a more typically observed BMI and age range. This would ensure that these findings, particularly those related to adverse effects, are generalizable to the average patient with rosacea.

## Author Contributions

H.V.: conception, drafting, writing, finalization. B.B.: drafting, writing, revisions. R.A.L.: revisions. G.R.: revisions. N.G.: conception, revisions. B.U.: revisions, finalization. All authors were involved in drafting the manuscript or critical revisions, gave final approval of the version to be published, and agreed to be accountable for all aspects of the work.

## Ethics Statement

The authors confirm that the ethical policies of the journal, as noted on the journal's author guidelines page, have been adhered to. No ethical approval was required as this is a review article with no original research data.

## Conflicts of Interest

B.U. is an employee of Mount Sinai and has received research funds (grants paid to the institution) from: Incyte, Rapt Therapeutics, and Pfizer. He is also a consultant for Arcutis Biotherapeutics, Bristol Myers Squibb, Castle Biosciences, Fresenius Kabi, Galderma, Janssen, Lilly, Pfizer, Primus Pharmaceuticals, Sanofi, and UCB. The other authors have no conflicts of interest.

## Data Availability

The authors have nothing to report.
